# Supplementary material for: Investigating the Effects of Diet-Induced Prediabetes on Skeletal Muscle Strength in Male Sprague Dawley Rats
Source: Int J Mol Sci. 2024 Apr 6;25(7):4076. doi: 10.3390/ijms25074076 (PMC11012655; doi:10.3390/ijms25074076)
Supplement: Supplementary file 1 [file ijms-25-04076-s001.zip › ijms-2926845-supplementary.pdf]

Table S1: Composition of the high fats high carbohydrates (HFHC) diet

Ingredient	Incl(%)	Mix(kg)
Maize	38.98	390.000
Palm Oil	20.99	210.000
Soya Full Fat	14.99	150.000
Wheat Gluten	6.50	65.000
Flour	6.00	60.000
Monodex	5.00	50.000
Sugar - White	5.00	50.000
Limestone	1.00	10.000
Dicalcium Phosphate	0.50	5.000
Vitamin Premix	0.35	3.500
Salt - Fine	0.30	3.000
Amino Acid - DL Methionine	0.30	3.000
Mineral Premix	0.10	1.000
	**100.01**	**1000.50**

Table S2: Nutritional value of the high fats high carbohydrates (HFHC) diet

Nutrient	Units	Actual
Dry Matter	g/kg	919.93
Metabolizable Energy	MJ/kg	15.86
Crude Protein	g/kg	151.27
AShreonine	g/kg	4.51
ASIsoluecine	g/kg	5.24
ASLysine	g/kg	6.54
ASMethionine	g/kg	4.86
ASryptophan	g/kg	1.30
ASstidine	g/kg	3.30
ASTSAA	g/kg	6.79
ASValine	g/kg	5.80
Fat	g/kg	250.46
Carbohydrate	g/kg	427.29
Fibre	g/kg	22.08
Ash	g/kg	26.31
Avl Phosphorus	g/kg	1.66

Calcium	g/kg	5.47
Total Phosphorus	g/kg	3.60

Table S3: Composition of fats, proteins and carbohydrates of the standard diet.

**Fats**	15 %
**Proteins**	30 %
**Carbohydrates**	35 %
**Other components**	20%
